# Drug Eluting Embolization Particles for Permanent Contraception

**DOI:** 10.1021/acsbiomaterials.2c00357

**Published:** 2022-06-24

**Authors:** Hannah VanBenschoten, Shan Yao, Jeffrey T. Jensen, Kim A. Woodrow

**Affiliations:** † Department of Bioengineering, University of Washington, 3720 15th Avenue Northeast, Seattle, Washington 98105, United States; ‡ Oregon National Primate Research Center, Oregon Health & Science University, 505 Northwest 185th Avenue, Beaverton, Oregon 97006, United States

**Keywords:** electrospinning, controlled release, drug delivery, sclerosing agents, permanent contraception

## Abstract

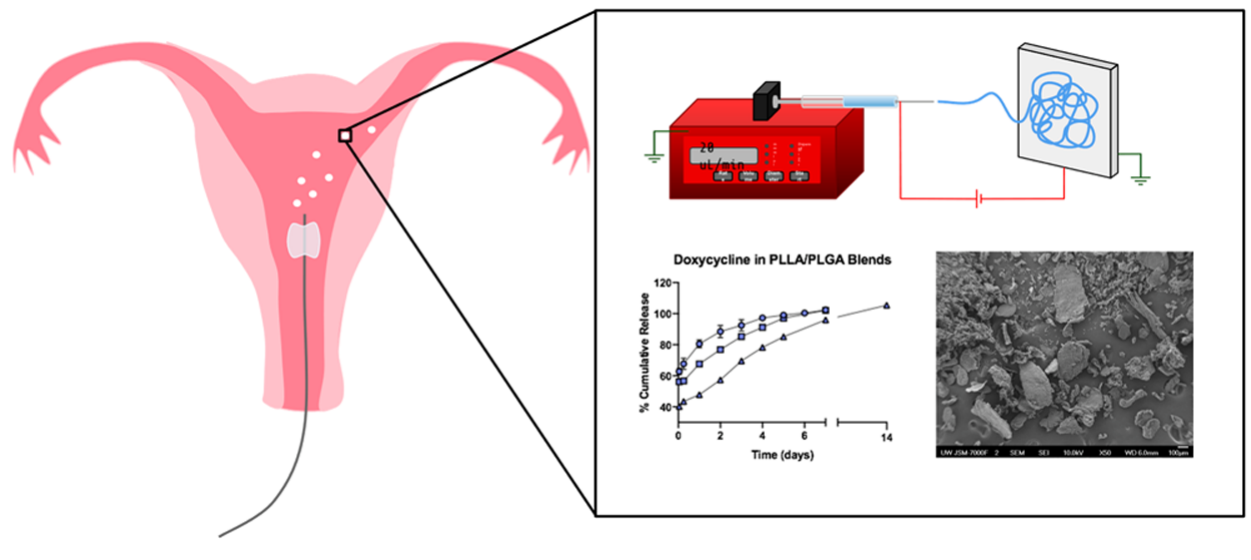

Medical technology that blocks the fallopian tubes nonsurgically could increase access to permanent contraception and address current unmet needs in family planning. To achieve total occlusion of the fallopian tube via scar tissue formation, acute trauma to the tubal epithelium must first occur followed by a sustained and ultimately fibrotic inflammatory response. Here, we developed drug-eluting fiber-based microparticles that provide tunable dose and release of potent sclerosing agents. This fabrication strategy demonstrates high encapsulation of physicochemically diverse agents and the potential for scalable manufacturing by utilizing free-surface electrospinning to generate material for fiber micronization. Manipulation of nanofiber formulation such as drug loading, drug hydrophobicity, polymer hydrophobicity, and crystallinity allowed for modulation of the sustained release properties of our fiber microparticles. We assessed various fibrous microparticle formulations *in vivo* using a newly developed and validated guinea pig model for contraception. We found that fiber microparticles with bolus release doxycycline effectively elicited acute trauma and those formulated with highly loaded phenyl benzoate caused sustained inflammation in the target organs. The demonstrated potency of these electrospun microparticles, as well as their embolic size and shape, suggests potential for proximal agglomeration and inflammatory activity in the fallopian tubes following transcervical delivery.

## 1 Introduction

Female permanent contraception is currently the most common form of birth control used worldwide, as it is the method of choice for almost a quarter of reproductive age women.^1^ This high demand for permanent birth control is met entirely by surgical intervention. Surgical approaches involve removing a segment or the entirety of both fallopian tubes or placing mechanically occlusive devices such as clips or bands. Both laparotomic and laparoscopic approaches require intraabdominal access, which exposes women to the myriad risks and drawbacks of any invasive surgery including adverse reactions to anesthesia, possible injury to local organs, bleeding, pain, and increased time and financial commitment.^2,3^ While surgical permanent contraception is highly effective and safe when quality medical facilities and trained clinicians are available, approximately 5 billion people worldwide lack access to safe and affordable surgical care. A safe and effective nonsurgical method of permanent contraception could help address the unmet need for contraception in women who have completed desired family size.

The fallopian tubes have a unique immunological and anatomical makeup that is intrinsically pro-healing to maintain tubal and reproductive function. Flow cytometric analysis of CD45^+^ leukocyte populations in the female reproductive tract shows that fallopian tube tissue is the most populated site of immune cells, the majority of which are CD3^+^ T cells and CD66b^+^ granulocytes.^4^ Fallopian tube physiology is characterized by extensive vascularization, high levels of mucosal secretions, and minimal luminal space surrounded by a muscular myometrium to restrict the entrance of contaminants, traits that collectively promote strong innate immunity. To overcome these barriers and elicit complete tubal occlusion, initial trauma to the fallopian tubes must be sufficient to provoke extensive epithelial damage followed by chronic inflammation that resolves in pathological scarring in lieu of re-epithelialization. This effect is mediated by the dose and duration of exposure to an antagonistic agent. The natural tubal occlusion that occurs following infection with sexually transmitted pathogens such as *N. gonorhoeae* and *C. trachomatis* illustrates this principle. Upon infection, the body’s innate immune system employs a mechanism of epithelial exfoliation to prevent colonization of harmful pathogens in the genital mucosa.^5^ Fallopian sequelae due to epithelial shedding and inflammation developed as survival mechanisms to combat systemic infection. Tubal occlusion prevents ascension of harmful pathogens into the peritoneal cavity, which is accessed by fimbriae at the distal end of the fallopian tubes. Interestingly, *N. gonorrheae* is capable of causing tubal occlusion after a single infection due to the potency of the lipoologosaccharide toxin released by the pathogen, which causes total epithelial desquamation.^6^ In contrast, *C. trachomatis* induces tissue damage but not total desquamation; therefore, multiple infections are required for occlusive tubal scarring to occur. Pursuant to the pathology of these infections, we hypothesize that the potency of an antagonistic agent can compensate for the duration of exposure, and vice versa. This paradigm potentiates the design strategy for novel nonsurgical permanent contraception.

Several current and prior methods of permanent contraception demonstrate this principle.^7−9^ While many surgical methods that use ligation or clipping function independently of time, other methods, such as thermally induced trauma via cautery or cryosurgery, require prolonged exposure to induce sufficient and irreversible tissue damage. Indeed, such injury is dampened by thermal buffering from extensive blood flow.^7^ Essure, a dynamically expanding nitinol and stainless steel microcoil that is placed transcervically into both fallopian tubes, was highly effective at eliciting fibrotic tissue encapsulation. This response was similarly time-dependent; clinical reports show that the rate of successful closure of the tubal lumen increased by over 50% when wear-time extended from a 4-week interval to an 8-week interval.^8^ Chemical sterilization approaches, which employ inflammatory agents that directly cause epithelial detachment or apoptosis leading to pathological scarring, show strong dose- and time-dependent occlusive effects. For instance, in a study evaluating the fibrotic effect of quinacrine pellet insertion in the fallopian tubes, the successful occlusion rate increased from 56% to 92% for pellet residency of 0–6 weeks to 7 or more weeks, respectively. The same study showed a distinct dose-dependent response, with the success rate increasing from 43% to 100% for a 100 mg versus 252 mg dose of the sclerosant.^9^ The Essure permanent implant and use of quinacrine pellets, though effective, serve only as lessons regarding features of successful occlusive techniques; both therapies lack full FDA approval due to severe side-effects. Though perhaps safer, instillation of liquid sclerosing agents, such as tetracycline and polidocanol, is limited by insufficient delivery to target tissue. Indeed, intrauterine administration often results in the target organ being exposed to less than 5% of the administered dose due to significant leakage.^9^ Thus, there remains a challenge to develop FDA-approved nonsurgical sterilization that targets the fallopian tubes and elicits fibrosis according to principles that govern successful occlusion.

While we know, from naturally occurring infections and prior clinical attempts, that successful nonsurgical tubal occlusion is a dose- and time-dependent process, a technique that integrates both features into an effective and clinically acceptable technology has yet to be developed. Additionally, there is currently no well-established or consistent platform to systematically investigate the optimal dose and time of exposure for a given antagonistic agent that could mediate effective tubal occlusion. To address these gaps, we aimed to develop a scalable platform to generate drug-eluting microparticles that can efficiently deliver sclerosing agents of diverse physicochemical properties to the fallopian tubes. We hypothesized that free surface electrospinning could be used as a scalable method for generating microparticles composed of polyester nanofibers that encapsulate highly loaded active agents and provide drug release at various time scales. We investigated polyesters, including poly(lactic-*co*-glycolic) acid (PLGA), polycaprolactone (PCL), and poly(l-lactic) acid (PLLA), to generate fibrous microparticles that could offer tunable release of encapsulated agents. These polymers biodegrade into readily metabolized products, which allows for transient residency in the fallopian isthmus and reduces potential migratory effects seen with other permanent contraceptive implants.^10^ Furthermore, evidence suggests that the release of acidic byproducts at the site of polyester degradation could enhance inflammation.^11,12^ We further hypothesized that polyester microparticles of inhomogeneous shape and size within a 100–300 μm diameter range may target and agglomerate in the fallopian isthmus into which they are perfused; this solid dosage modality can therefore deliver active agent while minimizing leakage associated with liquid-based treatments.^40−42^ Thus, our aims were to develop microparticles that (1) achieve high encapsulation efficiency of physicochemically diverse sclerosing agents, (2) can be processed into microparticles of target size and shape, and (3) can release encapsulated agents on a variety of time scales, from bolus release to investigate acute tissue responses to sustained release (>30 days) to assess long-term treatment outcomes. To test our hypothesis that these particles could elicit precursors to tubal occlusion, we validated a guinea pig model for permanent contraception and found that polyester fibers enhance acute inflammation, hemorrhage, and long-term fibrous capsule formation at the utero-tubal junction. These *in vivo* findings, along with the design space established by our versatile and scalable biomaterial fabrication approach, potentiate this method as a suitable alternative to nonsurgical permanent contraception methods previously under investigation.

## 2 Materials and Methods

### 2.1 Materials

Poly(d,l-lactic-*co*-glycolic) acid (PLGA) with a 50:50 LA:GA ratio, acid termination, and an inherent viscosity of 0.55–0.75 dL/g, and poly(l-lactic) acid (PLLA) with ester termination and an inherent viscosity of 0.90–1.20 dL/g in CHCl_3_ were purchased from Lactel Absorbable Polymers (Birmingham, AL, USA). Polycaprolactone (PCL) with an average molecular number (*M*_n_) of 80 000 Da, poly(d,l-lactide) (PDLLA) with an average molecular weight (*M*_w_) of 21 000 Da and an acid termination, and poly(vinyl alcohol) (PVA) with an average *M*_w_ of 105 000 Da were purchased from Sigma-Aldrich (St. Louis, MO, USA). Doxycycline hyclate was purchased from MP Biomedicals, LLC (Santa Ana, CA), and doxycycline hydrochloride (HCL) was purchased from Research Products International (Mount Prospect, IL, USA). Phenol and nonaethylene glycol monodecyl ether (Polidocanol) were purchased from Sigma-Aldrich. Phenyl benzoate was purchased from Alfa Aesar (Tewksbury, MA, USA). The solvents hexafluoroisopropanol (HFIP), 2,2,2-trifluoroethanol, chloroform, and dimethyl sulfoxide (DMSO) were purchased from Oakwood Laboratories (Wayne County, MI, USA), Sigma-Aldrich, Avantor Performance Materials (Bridgewater, NJ, USA), and VWR (Randor, PA, USA), respectively. Veterinary gelatin capsules (size 9, batch #VC191168) were purchased from Torpac Inc. (Fairfield, NJ, USA). Cremophor A25 was purchased from Sigma-Aldrich. High-performance liquid chromatography (HPLC) grade acetonitrile, trifluoroacetic acid, and water were obtained from Fisher Scientific (Pittsburgh, PA, USA). Dulbecco’s phosphate buffered saline (DPBS) was purchased from Mediatech Inc. (Central Valley, PA, USA).

### 2.2 Drug Treatment of Tissue Explants

Two fallopian tubes were obtained from an adult female *M. Mulatta* (hybrid, 12.5 y/o) rhesus macaque via the Washington National Primate Research Center Tissue Distribution Program. IACUC approval was not needed for *ex vivo* experimentation. Fresh fallopian tubes were collected in chilled Dulbecco’s modified Eagle medium with Nutrient Mixture F12 (DMEM:F12, Thermo-Fisher Scientific, Waltham, MA, USA), supplemented with 10% fetal bovine serum (FBS) (Gemini Bio-Products, Sacramento, CA, USA). Fallopian tubes were dissected to remove adventitial tissue and 12, 3 mm cross-sectional biopsy punches were taken from the ampulla of each tube, totaling 24 biopsy punches. Biopsies were placed in a 24 well plate and 500 μL of warmed DMEM:F12-FBS media supplemented with no drug, 1 μg/mL lipopolysaccharide (LPS), doxycycline hyclate in a 1%, 5%, or 10% (mg/mL) dose, or phenol in a 1%, 5%, or 10% (mg/mL) dose was added to each biopsy in triplicate. Samples were incubated at 37 °C for 24 h. After 24 h, media were removed from each sample and centrifuged at 10 000 rpm for 5 min to remove cellular debris. Tissues were washed twice with PBS; two biopsies for each condition were placed a fresh 24-well plate with 400 μL of warmed DMEM:F12-FBS. Then 80 μL of CellTiter Blue (Promega, Madison, WI, USA) was added to each biopsy and to three wells containing only media and incubated at 37 °C. After 4 h, 100 μL samples of media were taken in triplicate from each biopsy incubated with CellTiter Blue and fluorescence was recorded at 560_Ex_590_Em_. The remaining biopsy from each condition was fixed in 20 mL of formalin for 24 h at 4 °C before being sent to the University of Washington Histology and Imaging Core for sectioning and hematoxylin and eosin (HE) staining. The extent of cell infiltration and percent of epithelial detachment were quantified using ImageJ (NIH) by tracing and measuring the path length of in-tact epithelium divided by the total epithelial length. This percentage was defined as the percent of attached epithelium; the opposite of which was the percent of detached epithelium.

### 2.3 Preparation of Electrospun Fibers

Polyester blends loaded with doxycycline and phenyl benzoate were generated via uniaxial needle electrospinning and selected formulations were fabricated on a Production Line 1S500U Elmarco Nanospider. Then 80:20 PLGA/PCL was made by dissolving PLGA and PCL in an 80:20 (w/w) ratio at 15% (wt/vol) in HFIP. PLLA/PDLLA blends were made by dissolving PLLA and PDLLA at 100:0, 75:25, and 50:50 (w/w) ratios at 15% (wt/vol) in a 1:1 (v/v) mixture of chloroform and 2,2,2-trifluoroethanol. PLLA/PLGA blends were made by dissolving PLLA and PLGA in 100:0, 80:20, 50:50, and 20:80 (w/w) ratios at 15% (wt/vol) in a 1:1 (v/v) mixture of chloroform and 2,2,2-trifluoroethanol. 80:20 PLLA/PCL was made by dissolving PLLA and PCL in an 80:20 (w/w) ratio at 15% (wt/vol) in a 1:1 (v/v) mixture of chloroform and 2,2,2-trifluoroethanol. PVA was made by dissolving PVA at 15% (wt/vol) in water and heating slowly. Polymers were allowed to dissolve in solvent overnight and drugs were added a minimum of 1 h prior to electrospinning. All polymer formulations were also electrospun in the absence of drug (blank fibers). For 80:20 PLGA/PCL, doxycycline was added at 20%, 40%, 60%, and 80% (w/w); for PLLA, doxycycline was added at 20%, 40%, and 60% (w/w); for PLLA/PDLLA blends and PLLA/PLGA blends (excluding 20:80 PLLA/PLGA), doxycycline was added at 20% (w/w). For 80:20 PLGA/PCL blends, phenyl benzoate was added at 20%, 40%, 60%, and 80% (w/w). For needle electrospinning, approximately 500 μL of polymer solutions were loaded into a 1 mL glass gastight syringe equipped with a 21 gauge stainless steel dispensing needle and set into a NE1000 precision syringe pump (New Era Pump Systems Inc., Farmingdale, NY, USA). Unless otherwise noted, solutions were pumped at a rate of 30–50 μL/min through a 13 kV electric field applied by a high voltage generator (Gamma High Voltage Research) between the needle and a grounded metal plate covered by a sheet of wax paper set 13–15 cm from the needle tip. PVA was electrospun at 18 kV and pumped at a rate of 5 μL/min. For electrospinning on the Production Line NS 1S500U Nanospider (Elmarco, Czech Republic), fiber mats were generated in static conditions using 20 mL of polymer/drug solution. A 0.6 mm orifice was used to coat 350 mm of a rotating wire set 200 cm from a metal collector covered by a sheet of wax paper. A 100 kV charge was applied between the coated wire and the collector for a total run time of 15 min. Collected fiber mats were dried in a fume hood overnight and stored in vacuum sealed bags until use or analysis.

### 2.4 Drug Loading and *In Vitro* Drug Release from Electrospun Fibers

Effective drug loading, termed “encapsulation efficiency”, was defined as the total amount of drug associated with the fibers relative to theoretical loading. To determine encapsulation efficiency of doxycycline and phenyl benzoate in electrospun fibers, each fiber type was cut into approximately 4 mg samples (*n* = 3); exact mass was recorded for each sample. Fibers were placed in 7 mL of DMSO and attached to a rotisserie shaker for 3 days or until fibers were completely dissolved. Then 200 μL of supernatant was collected for HPLC analysis. Drug content was quantified with a Shimadzu Prominence LC20AD UV-HPLC system equipped with a Phenomenex Luna C18 column (250 × 4.6 mm^2^, 5 μm) and LC Solutions software. Doxycycline was detected with a mobile phase of HPLC grade water and acetonitrile (75:25) supplemented with 0.1% trifluoroacetic acid, eluted at an isocratic flow rate of 1 μL/min for 15 min and an injection volume of 20 μL. Column oven temperature was 30 °C. Doxycycline standards were prepared in DMSO with a linear range from 0.001 to 1 mg/mL, detection at 265 nm, and a retention time of 7.2 min. Phenyl benzoate was detected with a mobile phase of HPLC grade water and acetonitrile (70:30) supplemented with 0.1% trifluoroacetic acid eluted at an isocratic flow rate of 1 μL/min for 15 min and an injection volume of 40 μL. Column oven temperature was 30 °C. Phenyl benzoate standards were prepared in acetonitrile with a linear range from 0.05 to 0.5 mg/mL, detection at 265 nm, and a retention time of 5.93 min. Encapsulation efficiency was determined by calculating the drug concentration in the supernatant of dissolved fibers using standard curves and dividing by theoretical drug content according to the following equation:
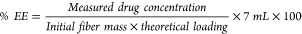
*In vitro* release of doxycycline and phenyl benzoate from electrospun fibers was carried out in 1X DPBS and in 1X DPBS supplemented with 1% Cremophor, respectively, in sink conditions. Sink conditions for doxycycline were determined using the published solubility of doxycycline in PBS (50 mg/mL). We determined the solubility of phenyl benzoate in 1% Cremophor by saturating an aliquot of PBS-1% Cremophor, centrifuging the supernatant upon equilibrium informed by visible precipitation of drug, and analyzing the saturation concentration on HPLC (2.4 mg/mL). Fibers were cut into 5 mg samples (*n* = 3), placed in 10 mL of release medium, and incubated in a 37 °C shaker. Spiked samples were prepared containing the theoretical amount of drug retained in each sample type dissolved directly in release medium with an equivalent mass of drug-free (blank) fiber (*C*_spiked_). Blank fibers in drug-free release medium were also prepared for each release study (*C*_blank_). At predetermined time points, 400 μL of solution was removed from each sample for analysis by HPLC and replaced with fresh media to maintain sink conditions. For *in vitro* release, the HPLC detection method for doxycycline was the same as that described above; doxycycline standard curves were prepared in 1X DPBS with a linear range from 0.1 to 2 mg/mL, detection at 265 nm, and a retention time of 8.3–8.5 min. The HPLC detection method for phenyl benzoate release was the same as described above, with the same standard curve for analysis prepared in acetonitrile. Because phenyl benzoate hydrolyzes to phenol and benzoic acid *in vivo* and in simulated *in vitro* release medium, standard curves were also prepared for pure phenol in acetonitrile following the same HPLC method as described for phenyl benzoate, with linearity established between 0.001 and 0.5 mg/mL, detection at 265 nm, and a retention time of 5.7 min. For quantification of drug release from fibers, the concentration of drug in solution at each time point was calculated from standard curves. For phenyl benzoate-loaded fibers, phenyl benzoate and phenol content were calculated independently from respective standard curves in acetonitrile and summed *post hoc* to capture the total hydrolyzable and hydrolyzed sclerosing agent content released. Therefore, “released phenol” refers to the total concentration of phenol and phenyl benzoate detected in release medium. Cumulative percent and cumulative dose of drug released from fibers was calculated according to the following equations:
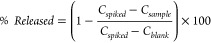


### 2.5 Micronization

Micronization was performed on nanofiber mats electrospun on the Elmarco Nanospider. Mats were cut into 1 in. by 1 in. squares and massed. Approximately 2 g of fibers was placed into a BlendTec MiniTwister Jar fashioned with a custom polystyrene lid that reduced jar volume to 8 oz. Rasping cycles of increasing intensity were used to generate microparticles, which were filtered through a 150 μm pore-size sieve (VWR), massed, and collected in a glass scintillation vial. Micronized particles were placed in a sterile tissue culture hood under UV light for 24 h. To confirm sterility, a 10 mg sample of drug-free particles was submerged in 40 mL of LB Broth (Sigma) and incubated at 37 °C. After 24 h, the sample was centrifuged to remove suspended particles and absorbance measured at 600 nm. The OD600 was compared to a sample incubated with no particles to verify the absence of bacterial growth. Sterile particles were then stored at 4 °C until use.

### 2.6 Polyester Blend Puncture and Adhesion Testing

Uniaxial puncture testing was performed with an Instron 5943 Mechanical Testing System (Instron) equipped with Bluehill 3 software. For puncture force analysis, polyester blends of 80:20 PLGA/PCL, PLLA, 80:20 PLLA/PLGA, 50:50 PLLA/PLGA, 20:80 PLLA/PLGA, and 80:20 PLLA/PCL prepared via uniaxial electrospinning were cut into 7/8′′ discs, massed, and measured for thickness using digital calipers calibrated to 0.01 mm. A 1′′ probe was attached to the load cell, and fibers were fixed onto the sampling platform above a 1/2′′ beveled hole. A 100N load was applied at a rate of 3 mm/s to the center of the sample until failure. The elongation to puncture was calculated by
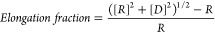
where *R* is the radius of fiber exposed to the beveled hole (1/2′′) and *D* is displacement of the probe from the point of contact to the point of fiber puncture. The puncture strength was calculated by
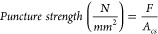
where *F* is the burst load required to puncture the fiber and *A*_*cs*_ is the cross-sectional area of the fiber at the edge of the beveled hole (thickness times circumference). This calculation normalizes for differences in sample thickness. The ratio of puncture strength to elongation fraction, herein called the relative puncture strength, was calculated from these equations. For mucoadhesion testing, PVA, 80:20 PLGA/PCL + 20 wt % doxycycline, 50:50 PLLA/PLGA + 20 wt % doxycycline, and 80:20 PLGA/PCL + 80 wt % phenyl benzoate fibers were cut into 7/8′′ discs, massed, and measured for thickness. Approximately 1 g of Type II mucin from porcine stomach (Sigma-Aldrich) was rehydrated in a Petri dish with 2 mL of deionized water to form a sticky paste. Fibers were fixed flat to the upper load cell and lowered to contact the mucus; upon contact, a 10N load was applied vertically to separate the specimen from the mucus until load returned to baseline.

### 2.7 Scanning Electron Microscopy

Representative micronized fibers composed of 80:20 PLGA/PCL loaded with 20 wt % doxycycline were spread in a monolayer on an SEM stub using conductive carbon tape. Samples were sputter coated with gold and palladium. Imaging was performed with a JSM-7000F SEM (JEOL Ltd.) at the Materials Science and Engineering Department at the University of Washington. An acceleration voltage of 10 kV and working distance 6 mm were used with magnifications of 50×, 250×, and 5000×. Fiber diameter was measured from the 5000× micrograph with ImageJ (NIH) software for a total of *n* = 30 fibers.

### 2.8 Laser Diffraction Particle Sizing

Microparticles generated with a 150 μm sieve were suspended at 5–10 mg/mL concentrations in a 5% surfactant (polidocanol) solution. Particle sizing was performed with a Horiba LA-960 Particle Size Analyzer (Kyoto, Japan) at the Materials Science and Engineering Department at the University of Washington. Deionized water with a real refractive index of 1.33 was used as the dispersion medium; electrospun fibers loaded with 20 wt % doxycycline and 80 wt % phenyl benzoate were sized with real refractive indices of 1.54 and 1.58, respectively. Circulation and agitation modes were set to 1, the system was debubbled, aligned, and blanked. Sample suspensions were added until transmittance was reduced by approximately 5%, and then measurements were taken in *n* = 5 replicates. Particle diameter was calculated based on a Mie Theory scattering algorithm and reported as the volumetric mean, D[4,3], and span is calculated according to the following equation:
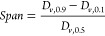
where *D*_*v*,0.9_ represents the diameter where 90% of the volumetric distribution lies below, the *D*_*v*,0.1_ where 10% of the volumetric distribution lies below, and the *D*_*v*,0.5_ represents the median diameter in which half of the distribution lies above and half lies below.

### 2.9 *In Vivo* Guinea Pig Procedures

Female guinea pigs housed at the Oregon National Primate Research Center were anesthetized prior to bilateral incision and flank laparotomy to expose the distal uterine horn on each side. We then injected 5% polidocanol foam (PF) or saline (1 mL) into the lumen of the distal uterine horns using a 23 gauge needle. Immediately following this, we made a small incision in the uterine wall to insert the gelatin capsules containing treatment into the distill horn.

We secured in place with a small loop of suture proximal to the capsule to close the space. Prior to the procedure, gelatin capsules were sterilized under a UV sterilizer (Dermologic-209) for 1 h. In a cell culture hood, sterile active materials were massed and packed into individual capsules using a capsule filling applicator. Uterine horn treatment groups included phenol (20 mg), phenyl benzoate (20 mg), silver nitrate (16 mg), empty capsules with 5% PF, doxycycline hyclate powder (10 mg) with 5% PF, 80:20 PLGA/PCL drug-free fibers (10 mg), 80:20 PLGA/PCL fibers +70 wt % doxycycline hyclate (20 mg) with and without 5% PF, 80:20 PLGA/PCL fibers +30 wt % doxycycline monohydrate (20 mg), and 80:20 PLGA/PCL fibers +80 wt % phenyl benzoate (20 mg). Animals were allowed to recover and monitored daily for their postoperative behavior, ability to eat and maintain weight, and stool quality. guinea pigs were euthanized and dissected 7 days and 30 days postsurgery. Immediately after necropsy, reproductive tracts were obtained and separated into upper ovary/horn and lower horn. Specimen were fixed in 4% paraformaldehyde, dehydrated in alcohol, and embedded with paraffin. Continuous 5 μm cross-sectional samples were taken and processed for HE staining. Histological features were scored based on predetermined criteria.

### 2.10 Ethics Statement

*In vivo* animal studies were approved by the Institutional Animal Care and Use Committee (IACUC) at the Oregon National Primate Research Center (Protocol #IP00003599). All animals were obtained and cared for in accordance with the IACUC guidelines.

### 2.11 Statistical Methods

Graphical results were expressed as the mean of each replicate ± standard deviation. One-way analysis of variance (ANOVA) using Tukey’s multiple comparison post-test was performed when comparing outcomes between groups, with statistical significance defined as *p* < 0.05 (*). Statistical analysis was performed on GraphPad Prism 8 software.

## 3 Results and Discussion

### 3.1 Sclerosing Agents Induce Cell Death, Epithelial Delamination, and Leukocyte Infiltration of Macaque Fallopian Tubes

Doxycycline and phenol have been used extensively as sclerosing agents for endovascular sclerotherapy and lymphatic cyst ablation^13,14^ but exhibit unique tissue responses that may be explained by their distinct mechanisms of action. The molecular mechanism of doxycycline as a tissue antagonist is speculated to involve inhibition of matrix metalloproteinases and suppression of vascular endothelial growth factor-induced angiogenesis, thereby preventing tissue repair and endothelial maintenance.^15^ The effect of doxycycline on epithelial layers has been studied using human bronchial epithelial cells, where it was shown to induce apoptotic pathways, necrosis, and cell detachment in a time- and concentration-dependent manner.^16^ Ultimately, this is correlated with in an inflammatory response that causes fibrosis and involution of endothelial-lined luminal structures. Phenol is a mildly acidic organic compound that has been used to treat hemorrhoids and varicose veins by causing hemorrhage of the endothelial submucosa resulting in obliteration of tissue.^14,17^ It has a direct toxic effect on human colonic epithelial cells cultured *in vitro*, consistent with the well-known ability of phenol to damage plasma membranes and inhibit cell growth.^18^ Here, we demonstrated the ability of both doxycycline and phenol to induce cell death and enhance leukocyte infiltration with unique effects on fallopian epithelial integrity.

We treated explant fallopian tubes obtained from a mature female *Rhesus Macaque* with varying concentrations of doxycycline and phenol to investigate the potential of these two active agents in initiating acute tissue trauma to fallopian epithelium. Phenyl benzoate is utilized in biomaterial formulations because it is a solid at room temperature and therefore able to form a solid dispersion while functioning as a sclerosant after dissolution and hydrolysis. Phenol and phenyl benzoate are considered analogous pharmaceutical compounds since the latter is an ester of phenol, which hydrolyzes in physiological conditions at a pH-dependent rate to form phenol and benzoic acid. In weak basic conditions, such as the fallopian tube, complete hydrolysis is expected to occur in under 30 min.^19,20^ Doxycycline was used in concentrations of 0.01 to 0.1% on monolayer epithelial cell cultures^16^ and is typically employed as a sclerosing agent in concentrations of 0.5 to 10%.^15^ Phenol has been assayed at concentrations of 0.1 to 0.5% in epithelial cell cultures^18^ and is injected in a 5% concentration in almond oil.^14^ As such, we chose to assay the inflammatory potential of 1%, 5%, and 10% solutions of each drug, concentrations that are 10-fold greater than those used to assay monolayer epithelial cell cultures. We chose to increase the concentration to account for the thickness of biopsy samples in comparison to single cell layer cultures and to recapitulate clinical doses. Lipopolysaccharide is a bacterial endotoxin commonly used to elicit a strong inflammatory response; at 1 μg/mL, it does not cause cell death but does stimulate the recruitment of leukocytes and release of inflammatory cytokines.^21^ As such, LPS was a positive control for assessing nontoxic inflammatory effects of the candidate sclerosants, whereas cell death, an important mechanism for inflicting acute trauma, was treated as a binary measurement in comparison to the media-treated control.

**Figure 1:**
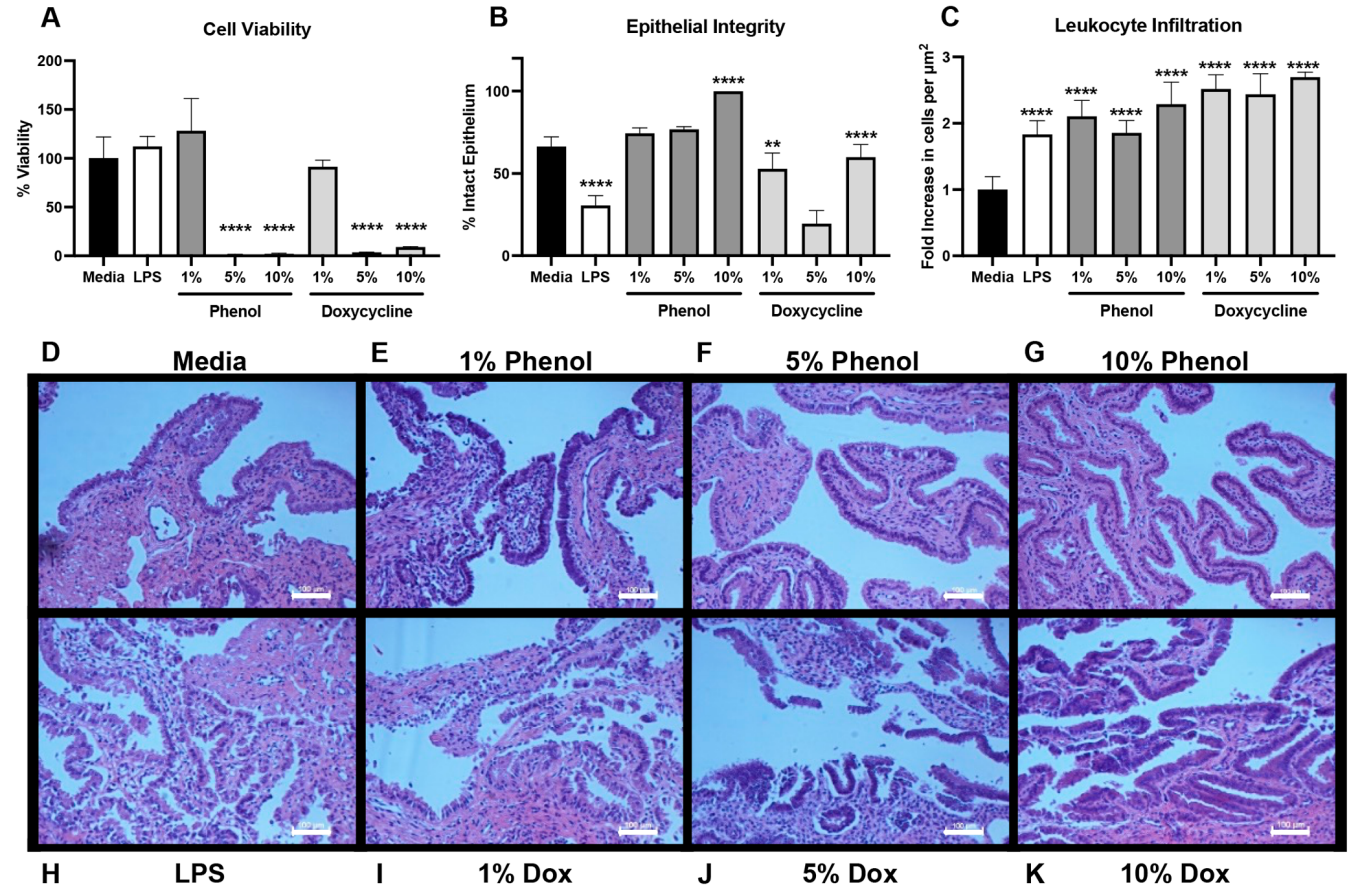
Treatment with sclerosing agents causes tissue damage and leukocyte infiltration. (A) Cell viability fraction of treated samples normalized to media treated samples. Percent expressed as the relative mean fluorescence ± standard deviation of *n* = 2 biopsies assayed with CellTiter Blue. (B) Percent of intact epithelium is expressed as the fraction of attached epithelium over the entire epithelial distance; results represent the mean ± standard deviation of *n* = 5 histological images taken at 10× magnification with widefield microscopy, capturing approximately 5000 μm of epithelial length per image. (C) Leukocyte increase expressed as fold-increase in cells per μm^2^ of lamina propria over the media-treated sample; results represent the mean ± standard deviation of *n* = 5 histological images taken at 20× magnification with widefield microscopy, capturing approximately 11 000 μm^2^ of tissue per image. (D–K) Fallopian tube biopsies cross-sectioned and stained with HE; images collected with 10× widefield microscopy. Scale bars represent 100 μm. (****) *p* < 0.0001. (**) *p* < 0.01.

After 24 h, biopsies treated with LPS, 1% phenol, and 1% doxycycline conditions showed no significant decrease in cell viability compared to media-treated controls (Figure 1A). Higher drug doses of 5 and 10% resulted in significantly reduced cell viability, indicating cytotoxicity of these treatments on explant biopsies after 24 h. We found that doxycycline results in a near dose-dependent decrease in epithelial integrity, visible in representative histological images (Figure 1I–K). This exfoliation was significantly greater than the untreated control (Figure 1B). In particular, the 5% doxycycline treatment condition resulted in severe destruction of the epithelium, causing approximately 80% of the columnar cell layer to detach from the lamina propria. Surprisingly, phenol results in a dose-dependent increase in the integrity of the epithelium and we observed marked preservation of the mucosal layer on the apical surface of the epithelium (Figure 1E–G). However, this observation may be an artifact of phenol acting as a fixative in conjunction with formalin. Indeed, the addition of 2% phenol to a 4% formaldehyde (formalin) solution has been shown to accelerate fixation processes and result in reduced tissue shrinkage and distortion.^22^ Despite differences in tissue morphology among the treatment groups, both drugs resulted in a significant increase in leukocyte infiltration consistent with that caused by LPS (Figure 1C). On the basis of the observed tissue effects of the sclerosants on fallopian biopsies, we conclude that doxycycline has an antagonistic effect on fallopian epithelial structures that results in cell apoptosis, detachment, and inflammation. Although phenol did not enhance epithelial delamination, it caused cytotoxicity and immune-cell infiltration. We therefore posit that phenol has interesting sclerosing potential based on its immune activation that warrants further investigation. Thus, both drugs represent promising candidates for evaluation of physiologically distinct chemical induction of time- and dose-dependent fallopian fibrosis.

### 3.2 Electrospun Polyester Blends Show High Encapsulation Efficiency and Processability for Micronization

Electrospun polyester blends were loaded with doxycycline and phenyl benzoate ranging from 20 wt % to 80 wt % drug loading. PLGA/PCL (80:20) and PLLA fibers loaded with doxycycline and phenyl benzoate show high encapsulation efficiency at all drug loadings (Table 1), with up to 77% and 73% encapsulation efficiency measured for the 80 wt % drug loading, respectively. Doxycycline could not be spun at 80 wt % in PLLA as it was insoluble in the electrospinning solvent at a concentration above the critical micelle concentration of the polymer. A general trend of decreasing encapsulation efficiency with increasing drug loading is observed for both active agents. This result is consistent with previous data showing that higher drug loading is typically associated with a greater fraction of drug being surface associated, which results in a relative reduction of encapsulated drug within the fiber matrix.^23^ Variations in polyester blend formulation had little impact on encapsulation efficiency (Table 2). Doxycycline loading at 20 wt % in PDLLA/PLLA and PLLA/PLGA blends achieved 83% to 93% encapsulation efficiency, despite differences in polymer crystallinity and hydrophobicity. Phenyl benzoate loaded at 20 wt % in 50:50 PLLA/PLGA achieved 72% encapsulation efficiency (not shown). We did not measure a significant difference in efficiency between the 20 and 80 wt % loading conditions. The high loading efficiency of electrospun material demonstrates the utility of this approach in formulating scalable biomaterials with minimal active agent loss compared to other particle fabrication approaches. Indeed, single and double emulsion particle formulation methods often fail to achieve higher than 10% efficiency in drug loading.^24,25^

**Table 1:** Drug Encapsulation in Formulations with Incremental Theoretical Loading^a^

theoretical loading (wt %)		20%	40%	60%	80%
doxycycline	80:20 PLGA/PCL	18.93% (94.64 ± 2.91)	34.96% (87.40 ± 1.79)	48.12% (80.20 ± 1.05)	61.58% (76.98 ± 1.24)
	PLLA	18.79% (93.79 ± 12.31)	39.42% (98.55 ± 5.54)	52.61% (87.69 ± 6.79)	
phenyl benzoate	80:20 PLGA/PCL	18.07% (90.34 ± 2.21)	34.48% (86.19 ± 2.03)	46.38% (77.30 ± 4.36)	58.62% (73.27 ± 1.60)

a Actual loading expressed as wt % and (mean encapsulation efficiency (%) ± standard deviation of 3 samples per condition).

**Table 2:** Drug Encapsulation (20 wt % Theoretical Loading) in Polyester Blends^a^

fiber blend	50:50 PLGA/PLLA	80:20 PLGA/PLLA	75:25 PLLA/PDLLA	50:50 PLLA/PDLLA
doxycycline	16.70% (83.50 ± 0.17)	17.00% (84.99 ± 10.84)	18.29% (91.45 ± 8.60)	18.00% (89.98 ± 14.99)

a Actual loading expressed as wt % and (mean encapsulation efficiency (%) ± standard deviation of 3 samples per condition).

**Figure 2:**
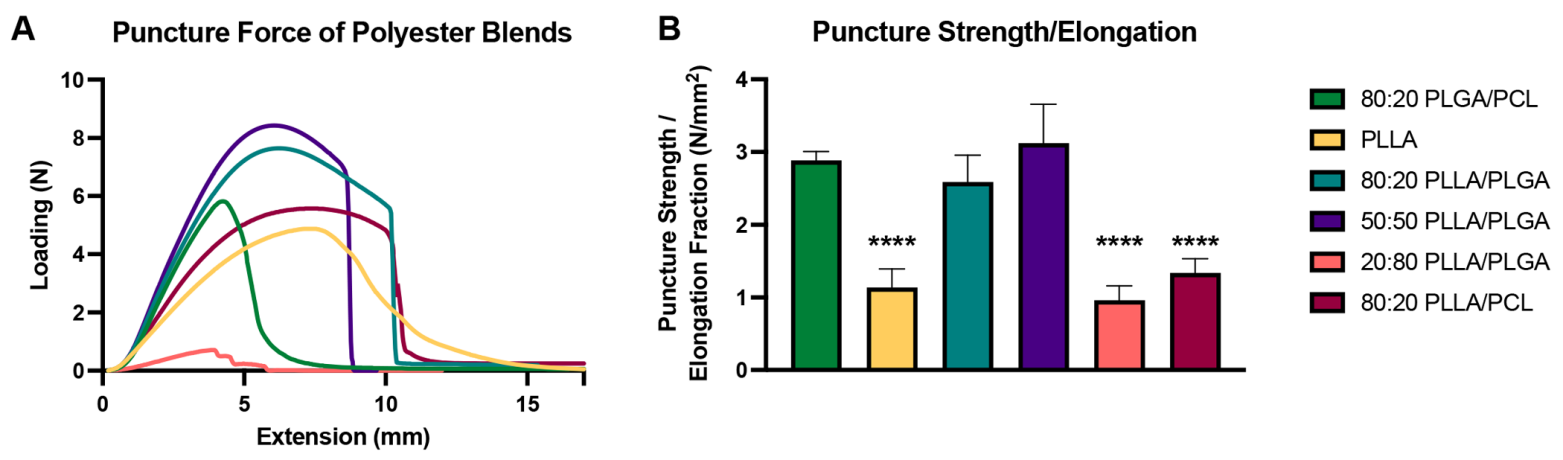
Puncture force of polyester blends informs micronizable formulations. (A) Puncture force of drug-free polyester blends generated via uniaxial electrospinning performed by a puncture test. (B) Calculated puncture strength per elongation fraction of polyester blends. Results presented as the mean ± standard deviation of tensile tests performed on at least *n* = 3 individual fiber samples per condition.

We micronized drug-loaded electrospun fibers to efficiently generate microparticles for transcatheter instillation and anatomical targeting. We first produced large nanofiber mats on a commercial-scale Elmarco NanoSpider and micronized the mats via a process similar in principle to traditional milling techniques, in which fiber comminution is the product of attrition and impact against steel blades and vessel walls.^26^ As this process is relatively low energy, mechanical properties of the electrospun fibers play a role in the success of micronization. High brittleness and minimal elongation before fractionation are desirable when impact is responsible for granulation. The combination of these properties can be described by material toughness. Radenbaugh et al. presented methods for characterizing material toughness via puncture test, which was shown to better resolve differences in the elongation property between materials than the tensile test.^27^ Prior experiments demonstrate that 80:20 PLGA/PCL electrospun fibers can be micronized with greater than 50% yield (data not shown), indicating promising processability characteristics of that blend. We measured the puncture strength divided by the elongation prior to puncture, herein called relative puncture strength, of candidate materials as a metric to identify polyester blends that could be successfully micronized. The burst load (maximum load before puncture), puncture strength, elongation fraction, and relative puncture strength of each blend are presented in Supplementary Table 1. Using the average of at least *n* = 3 puncture loads (N) versus probe extension (mm) (Figure 2A, Supplemental Figure 1), we found that 80:20 and 50:50 PLLA/PLGA blends have relative puncture strengths that are not significantly different from 80:20 PLGA/PCL fibers (Figure 2B). PLLA alone showed a more than 2-fold reduction in relative puncture strength; the dynamic behavior upon extension showed a protracted necking period followed by relaxation, rather than fractionation, which indicates highly ductile behavior (Figure 2A). Indeed, micronization of PLLA was unsuccessful and resulted in compression of fiber segments rather than comminution. Though 80:20 PLLA/PLGA exhibited a comparable relative puncture strength to that of 80:20 PLGA/PCL, its micronization was unsuccessful as well, perhaps due to elongation prior to fractionation. The 50:50 blend of PLLA/PLGA, which exhibited the highest relative puncture strength, was amenable to successful micronization with a yield of 8.5%, which was similar to the positive control. Differences in micronization success were likely dictated in part by the glass transition temperature (*T*_g_) and melting temperature (*T*_m_) of electropsun fibers. The *T*_g_ of PLGA and PLLA (Supplementary Table 2) and their blends are in the range of 40 to 60 °C, while the *T*_m_ of PCL is around 60 °C with a very low *T*_g_.^31,47−50^ Because of friction during the micronization process, it is possible that electrospun fibers experiences an increase in temperature nearing the *T*_g_ of PLGA and PLLA and near the *T*_m_ of PCL. This would have caused the material to change from its glassy, brittle state to an elastic and more ductile state, which would have impeded micronization. Informed by mechanical properties of each nanofiber formulation, we rationally selected blends that could be successfully micronized.

**Figure 3:**
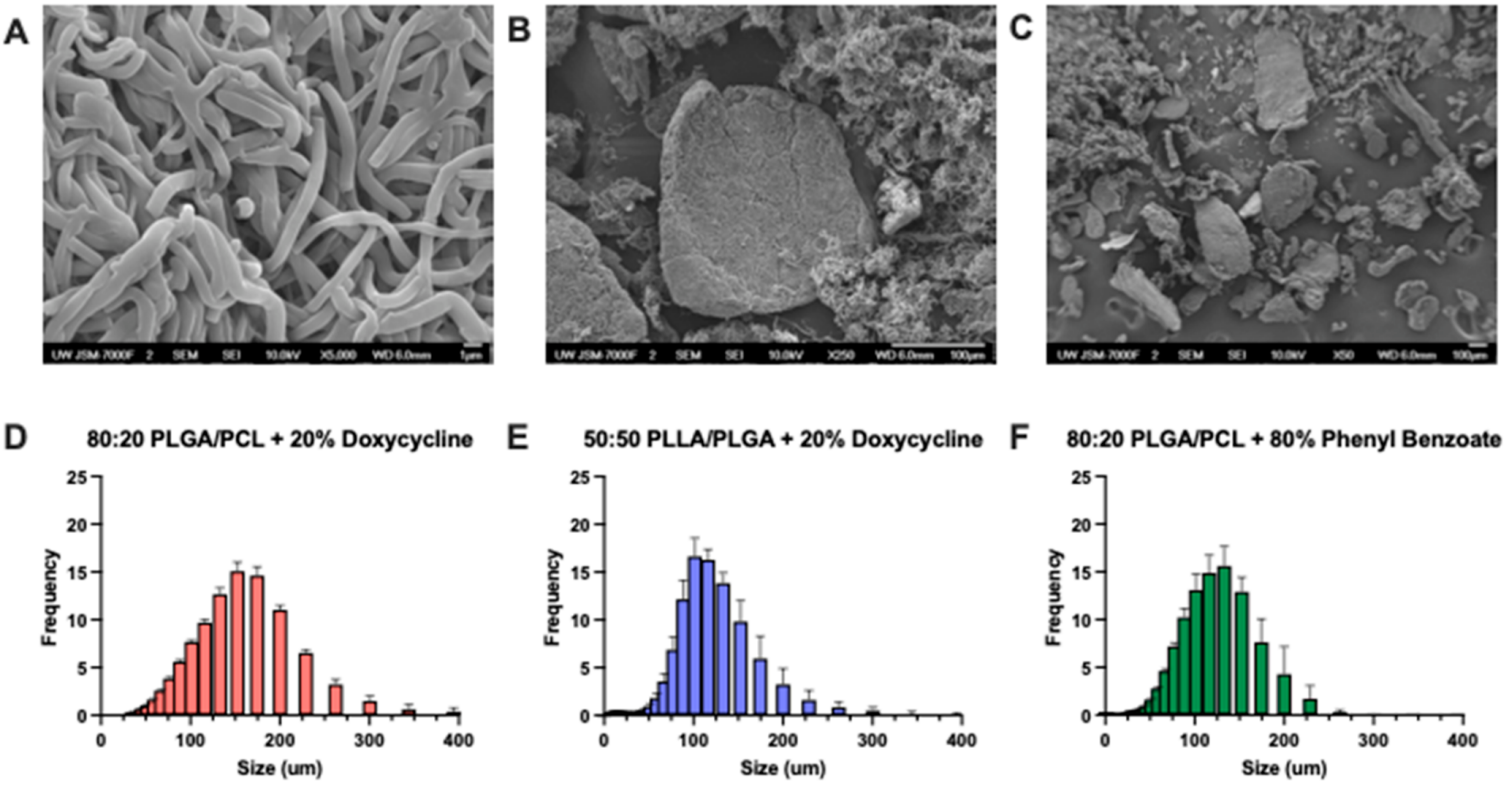
Fiber micronization generates particles of inhomogeneous shape with a Gaussian size distribution. Scanning electron microscopy of micronized 80:20 PLGA/PCL fibers loaded with 20% (w/w) doxycycline taken at (A) 5000×, (B) 250×, and (C) 50×. Volumetric particle size distribution of (D) 80:20 PLGA/PCL with 20% (w/w) doxycycline particles, (E) 50:50 PLLA/PLGA with 20% (w/w) doxycycline particles, and (F) 80:20 PLGA/PCL with 80% phenyl benzoate particles. Amplitude is represented as the mean ± standard deviation of 5 repeated measurements for each particle type on the laser diffraction particle sizer.

We used scanning electron microscopy and laser diffraction particle sizing to verify the success of fiber micronization. SEM images of sub-150 μm micronized particles achieved the desired heterogeneous size and shape (Figure 3A–C). According to prior studies evaluating the occlusive propensity of commercially available embolization particles, shape heterogeneity is generally considered to enhance macroparticle aggregation.^28^ Mesh fibers maintained a dense matrix with an average fiber diameter of 0.802 ± 0.133 μm (Figure 3A). In comparison, Carson et al. reported an average fiber diameter of 1.0–1.6 μm for PLGA/PCL blends that were not micronized but prepared with the same electrospinning parameters;^31^ therefore, we assume that micronization does not significantly effect fiber microarchitecture. Microparticles of PLGA/PCL (80:20) loaded with 20 wt % doxycycline had an average diameter of 141.47 μm; 50:50 PLLA/PLGA + 20 wt % doxycycline particles had an average diameter of 107.40 μm; and microparticles of PLGA/PCL (80:20) loaded with 80 wt % phenyl benzoate had an average diameter of 111.17 μm (Figure 3D–F). Alternate methods of particle synthesis are limited to generating particles of nanometers to several micrometers in diameter. These approaches fail to reach the 100–300 μm size specification for fallopian tube embolization.^11^ Using our micronization approach, all three particles exhibited favorable size distributions where the average diameter was below that of the guinea pig fallopian isthmus, allowing for entry and penetration.

Particulate formulation at this scale has many therapeutic advantages. The industry standard for commercial embolization particles describes particle size ranges that span 200 μm, which has been proven to allow for site-specific accumulation.^29^ The targeted portion of the human oviduct, called the isthmus, has a 1 mm to 0.1 mm diameter lumen with numerous longitudinal mucosal folds and extensive secondary folds that collectively fill the lumen.^7^ We propose that particles calibrated to match this luminal diameter, such as the 100–300 μm size range of our microparticles, may effectively aggregate within mucosal folds. Furthermore, the isthmus is the primary site of mucin production in the fallopian tubes.^30^ Therefore, the mucoadhesive property of these polyester blends provides further evidence for the potential of anatomically targeted particle aggregation. Indeed, PLGA/PCL and PLLA/PLGA fibers loaded with doxycycline and phenyl benzoate showed equivalent or significantly increased tensile adhesion to rehydrated porcine mucin compared to nanofibers composed of poly(vinyl alcohol) (PVA), a highly mucoadhesive polymer (Supplementary Figure 2). Therefore, our electrospun microparticles have potential for proximal aggregation in the human fallopian isthmus via intrauterine transcervical catheter delivery due to their Gaussian granulometric distribution that matches anatomical specifications of target tissue, inhomogeneity in shape, and mucoadhesive surface properties.

### 3.3 Microparticles Demonstrate Tunable Drug Release *In Vitro*

**Figure 4:**
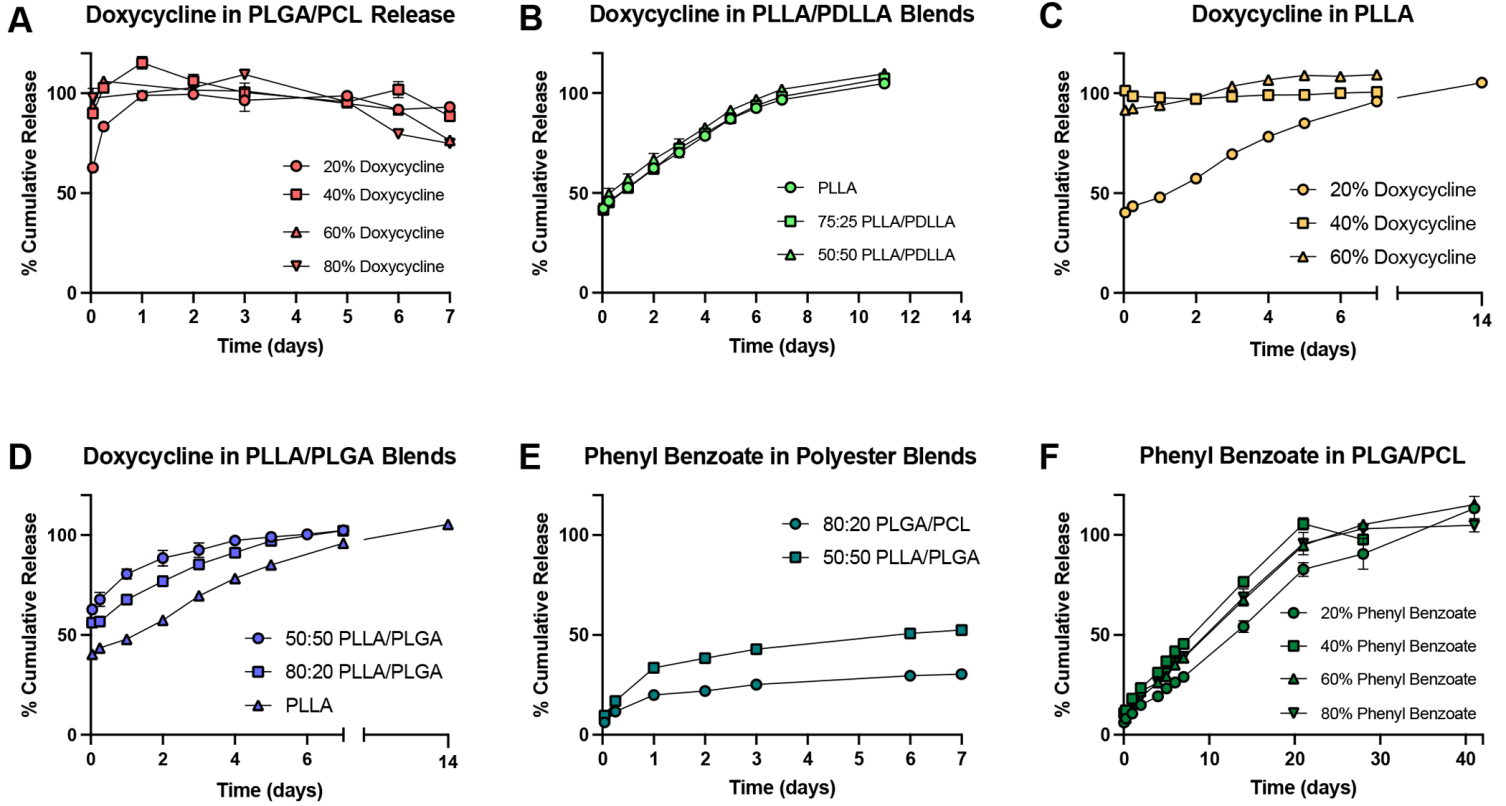
Fiber blends containing varied drug type and loading show tunable release profiles. *In vitro* drug release profile of (A) doxycycline from 80:20 PLGA/PCL fibers, (B) 20 wt % doxycycline from PLLA/PDLLA fiber blends, (C) doxycycline from PLLA fibers, (D) 20 wt % doxycycline from PLLA/PLGA blends, (E) 20 wt % phenyl benzoate from 80:20 PLGA/PCL and 50:50 PLLA/PLGA fibers, and (F) phenyl benzoate from 80:20 PLGA/PCL fibers. Results presented as the mean ± standard deviation of *n* = 3 fiber samples per condition, taken over a one to six week time course.

We formulated polyester blends to achieve release of encapsulated sclerosing agents ranging from burst release within 24 h to sustained release out to several weeks. These release kinetics were selected to recapitulate both the acute and chronic wound healing that has been observed to precipitate occlusion of the fallopian tubes. Thus, one burst release and one sustained release formulation were sought for this purpose. Previous work has shown that the addition of PCL to PLGA fibers affords greater tunability of drug release of tenofovir (TVF), a water-soluble antiretroviral.^31^ Therefore, we selected an 80:20 PLGA/PCL blend, which demonstrated sustained release of TVF, for electrospinning doxycycline hydrochloride (HCl, herein referred to as doxycycline), which is similarly water-soluble in its salt form. We investigated the effect of high drug loading on release kinetics from 80:20 PLGA/PCL fibers loaded with 20 wt % to 80 wt % doxycycline (Table 3, Figure 4A). For fibers loaded with 20 wt % drug, an initial burst release of 60% of encapsulated drug was followed by sustained release for 24 h. However, for each fiber formulation containing 40 wt % doxycycline or higher, approximately 90% to 100% of doxycycline burst released within the first hour. Doxycycline HCl is hydrophilic with a partition coefficient of −1.9 and may become more surface associated during the electrospinning process, resulting in rapid drug partitioning (release) into the aqueous medium. The relative ratio of polymer matrix to doxycycline appears to affect the partitioning kinetics, wherein a formulation of 80 wt % polymer matrix dampens diffusion for 24 h while formulations composed of 60 wt % or less in polymer mass fail to offer any sustained release. Thus, highly hydrophilic doxycycline is ideally formulated into burst release particles to elicit initial acute inflammation.

**Table 3:**
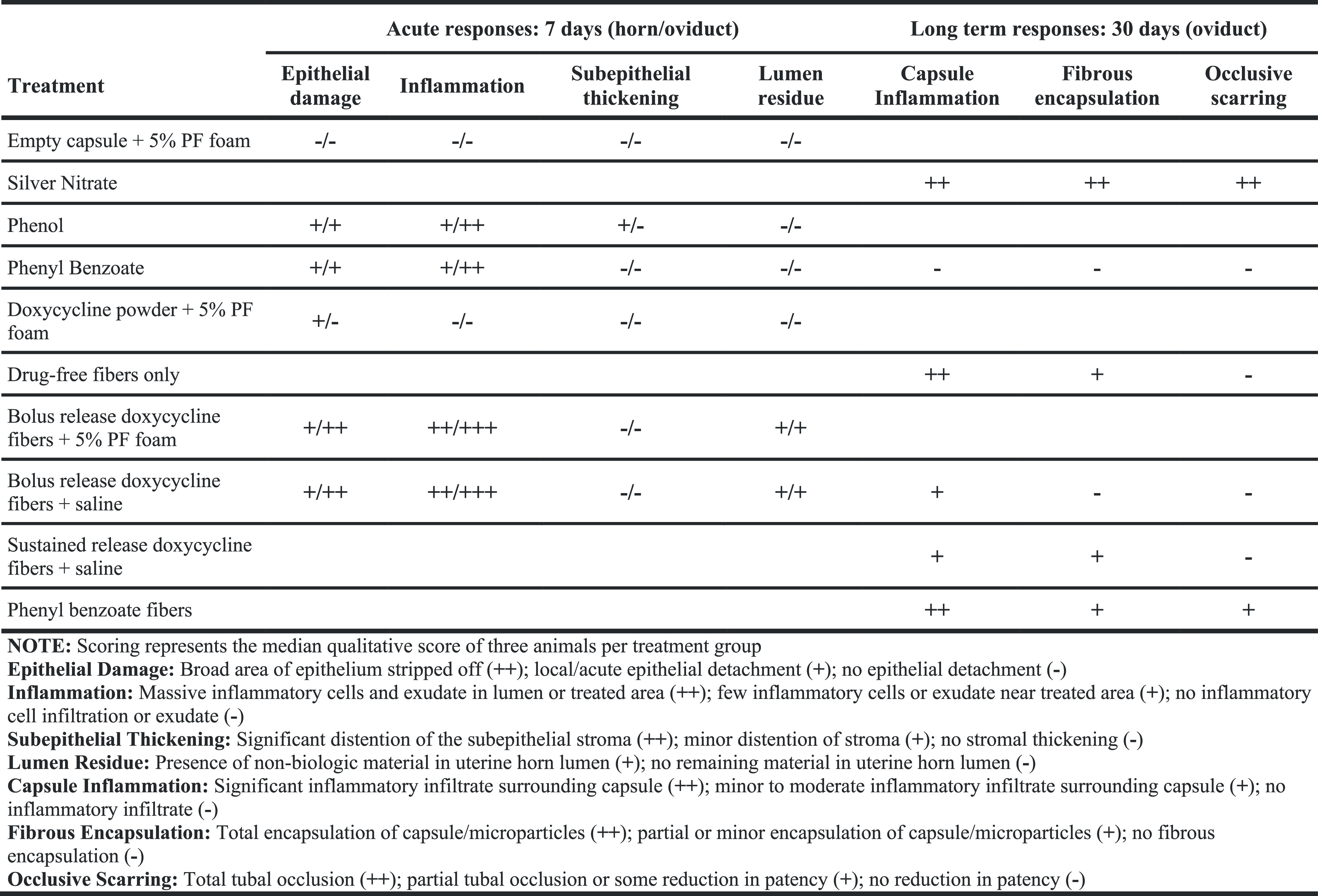
Guinea Pig Oviduct/Uterine Horn Acute and Long Term Treatment Outcomes

In addition to burst release particles formulated with PLGA/PCL, we sought to investigate the more crystalline PLLA to achieve sustained release of doxycycline. To directly investigate the effect of polymer crystallinity, we electrospun fibers containing 20 wt % doxycycline in blends of PLLA with its amorphous isomer poly-d,l-lactic acid (PDLLA) (Table 3). There was no difference in doxycycline release between the three PLLA blends; thus, the higher capacity for water penetration into racemic PDLLA chains did not increase drug release as expected (Figure 4B). However, PLLA achieved sustained release of doxycycline loaded at 20 wt % for 7 days. We next sought to investigate the effect of higher loading on the release of doxycycline from PLLA. We observed that PLLA fibers loaded with over 20 wt % doxycycline exhibited burst release of approximately 100% of encapsulated drug (Figure 4C), which may be due to higher amounts of surface associated drug. To confirm the relative effect of PLLA to PLGA fiber content on drug release kinetics, we electrospun blends of PLLA/PLGA (100:0, 80:20, and 50:50) fibers loaded with 20 wt % doxycycline (Table 3). As expected, we found that increasing PLLA content increased sustained release that could be tuned incrementally from 4 to 14 days (Figure 4D). Thus, the increased hydrophobicity of PLLA compared to PLGA permitted effective tuning of the release of water-soluble doxycycline with fine temporal resolution. These doxycycline formulations sustained release up to two-weeks, which may be sufficient to elicit a chronic response *in vivo*.

To generate material that could offer sustained release with a higher dose of encapsulated agent, we formulated particles with phenyl benzoate, which is highly hydrophobic compared to doxycycline and has a logP partition coefficient of 3.59. Interestingly, 20 wt % phenyl benzoate exhibited slower release from 80:20 PLGA/PCL fibers than from a PLLA-based fiber blend (Figure 4E). After 7 days, approximately 22% more phenol was detected from 50:50 PLLA/PLGA fibers than 80:20 PLGA/PCL fibers. Accordingly, 80:20 PLGA/PCL fibers were assayed for *in vitro* release of 20 wt % to 80 wt % phenyl benzoate until 100% release was observed. This fiber formulation showed sustained release of all drug loading conditions for up to 4 weeks, representing a promising candidate for release of a high drug dose from electrospun fibers over an extended period (Figure 4F). Previous research demonstrates that the degradation of PLGA/PCL blends is insignificant at time scales relevant to these release rates; therefore, we expect that water penetration and drug diffusion are primarily responsible for drug release.^31,32^ The release kinetics demonstrated by polyester microparticles may be advantageous in contributing to a persistent inflammatory state that recapitulate the timeline of physiological events necessary for tubal occlusion. This platform is therefore useful to probe dose- and time-response to pro-inflammatory and pro-fibrotic agents.

### 3.4 Validation and Sensitivity Assessment of Guinea Pigs as a Model for Permanent Contraception

Both acute inflammation and long-term antagonistic activity are critical to eliciting permanent fibrotic tissue formation for tubal occlusion.^33^ Here, we used a guinea pig model to investigate the activity of sclerosing agents and the impact of polyester fibers as both a delivery vehicle for burst and sustained release of drug as well as the antagonistic materials themselves. Significant research emphasis in contraception, including permanent contraception, has been placed on nonhuman primate (NHP) models due to similarities with human reproductive anatomy and physiology.^34^ Rodent models, including guinea pigs, have been used in contraceptive studies to identify molecular contraceptive targets; however, their use has not been reported for studying fallopian occlusion. We selected guinea pigs for these procedures as their uterine horns are 3–4-times larger in diameter than mice and rats (6–8 mm versus 2 mm), allowing for easier instillation of treatments.^35,36^ Here, we sought to identify if guinea pigs could be used as a model to assess tubal occlusion and then applied this model to assess our treatment regimens for this purpose.

Guinea pigs have anatomically distinct uterine horns that allow administration of different treatment conditions to the left and right horns in the same animal. We took histological samples in the proximal region of the uterine horn directly below the suture location as well as the distal region above the suture location where the uterine horn meets the oviduct. These anatomical locations are referred to as the uterine horn and oviduct, respectively. We qualitatively assessed the acute tissue response 7-days after various treatments according to epithelial detachment, inflammation, subepithelial thickening, and the presence of residual material in the uterine horn lumen. Long-term affects were characterized 30 days after treatment based on inflammation remaining near capsule placement, fibrous encapsulation of lumenal contents, and occlusive scarring (Table 3). No adverse side effects of this treatment were observed during the 7- or 30-day study periods. All animals had normal postprocedure behavior, ate, and maintained weight with an average change in weight between surgery and necropsy among all treatment groups of 1.68 ± 11.7%. No other parameters of well-being were collected in this study; future iterations of evaluating this treatment must involve characterizing its long-term side effects.

**Figure 5:**
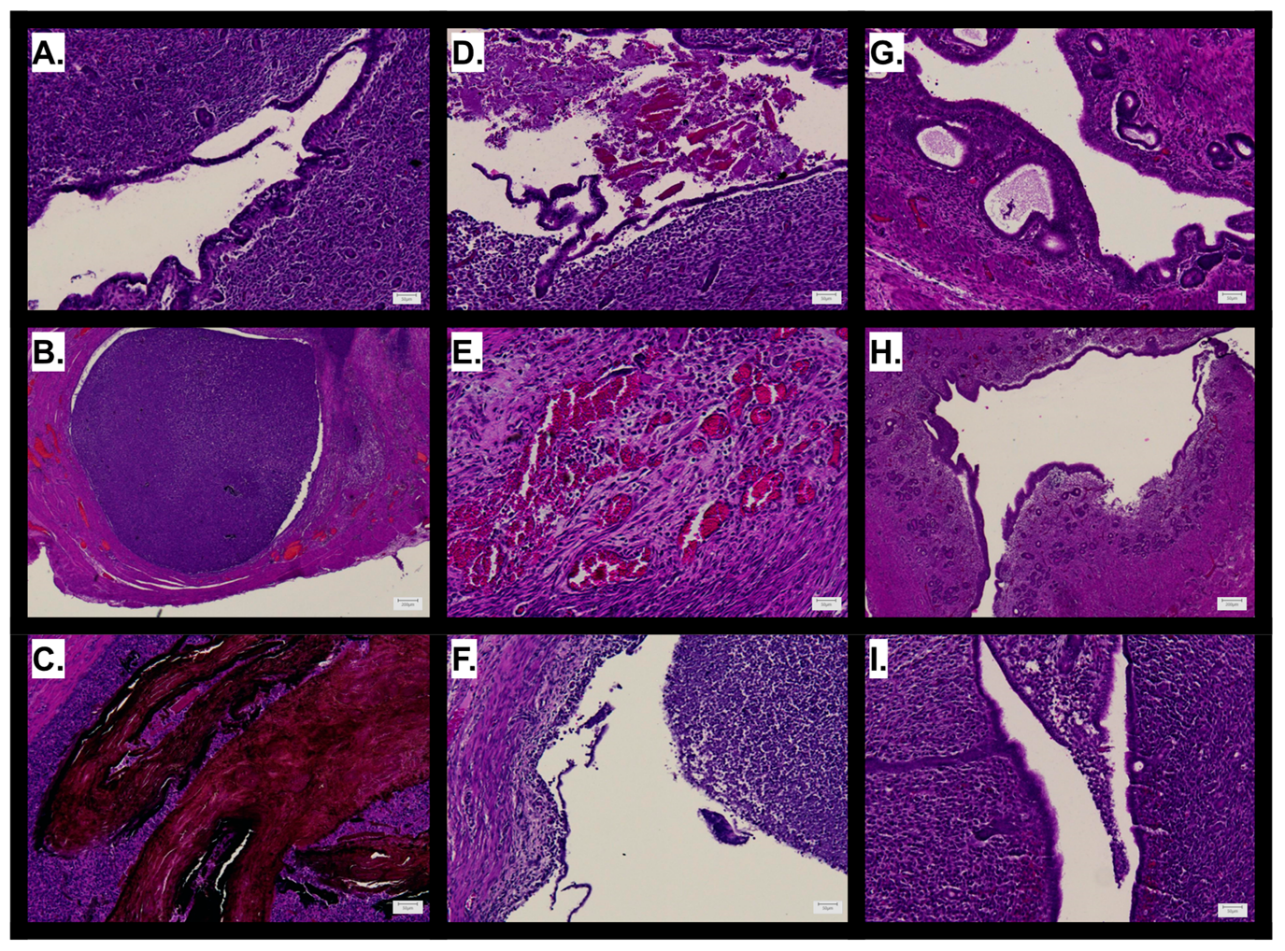
Histological features of guinea pig oviducts and uterine horns after treatment with active agents. Representative images from uterine horns treated with (A) empty gelatin capsule instilled with 0.5 mL of 5% PF for 7 days; (B, C) 16 mg of silver nitrate instilled with saline for 30 days; (D, E) 20 mg of phenol for 7 days; (F) 20 mg of phenyl benzoate instilled with saline for 7 days; (G, H) 20 mg of phenyl benzoate instilled with saline for 30 days; (I) 10 mg of doxycycline powder instilled with 0.5 mL of 5% PF for 7 days.

To understand the utility and constraints of the guinea pig model for evaluating sclerosant agents for tubal occlusion, we treated uterine horns with active agents alone including treatments comparable to published trials in higher order animals. We assessed the long-term effects of silver nitrate, a highly potent sclerosant, in the guinea pig model as a positive control. Silver nitrate was the first chemical agent tested for nonsurgical sterilization, and subsequent studies have shown that silver nitrate at low doses is superior to phenol at inducing long-lasting tubal occlusion in primates.^37,38^ Despite its potential, silver nitrate is considered highly toxic to skin and mucosal surfaces and thus is not FDA approved for use in humans and used here only as a positive control.^39^ Indeed, we observed extensive fibrotic tissue and near complete reduction of potency caused by occlusive scarring in the uterine horn and oviduct after treatment with 16 mg of silver nitrate (Figure 5B,C). Therefore, the guinea pig model recapitulates the phenotype of tubal occlusion when exposed to highly potent sclerosants such as silver nitrate and is valid for assessing permanent contraception at this potency threshold.

We next evaluated the sensitivity of this model relative to the tissue response in NHPs treated with less potent sclerosing agents than the positive control. We selected polidocanol foam as it requires repeated dosing in rhesus macaques to elicit tubal damage and blockade; therefore, it is known to cause occlusion but is less potent than silver nitrate.^40^ Indeed, varied results have been seen in single treatment trials in rodents which has motivated addition of more potent agents with longer residency to elicit more consistent occlusion.^41^ Here, we instilled a single dose of 5% polidocanol foam in empty gelatin capsules to improve residency time in the uterine horns. We minor epithelial detachment in one of three treated horns but no acute tissue trauma as seen with the silver nitrate (Figure 5A). In guinea pigs, polidocanol foam at this dose alone does not cause a robust tissue response and gelatin capsules do not sufficiently enhance residence time and increase the local effect of polidocanol. The guinea pig horns are more recalcitrant than NHP fallopian tubes to the effects of polidocanol foam, which makes the model appropriate for identifying highly potent sclerosants.

As experimental controls for our materials, we characterized the response of guinea pig uterine horns to the active agents used in our formulations (neat phenol, phenyl benzoate, and doxycycline). Phenol resulted in extensive epithelial damage and inflammation in addition to appearance of unknown lumenal contents that may suggest early collagen deposition or hemorrhage (Figure 5D). Tissue surrounding the affected lumen shows vascular hyperemia, further evidence of acute damage and inflammation (Figure 5E). *In vivo*, phenyl benzoate resulted in extensive purulent discharge into the fallopian lumen containing a mass of inflammatory cells (Figure 5F). As phenyl benzoate was instilled in its crystalline form, we anticipated slower degradation and thus examined its long-term effect on the oviduct. In contrast to its acute effects, phenyl benzoate resulted in minimal long-term tissue responses that would indicate persistent irritation (Figure 5G,H). The final drug-only treatment we tested was the effect of adding doxycycline powder to 5% polidocanol foam. Previous trials in baboons have shown that 5% PF supplemented with a 100 mg bolus dose of doxycycline causes a significant reduction in tubal patency marked by complete obliteration of the tubal epithelium and replacement with collagen-III.^42^ However, we observed in the guinea pig uterine horn model that this treatment resulted in minimal epithelial damage and inflammatory exudate in only one uterine horn (Figure 5I). Observing little tissue change or inflammation in guinea pig uterine horns due to this treatment also supports that a higher threshold of acute trauma is needed for an observable effect, thereby demonstrating the rigor of this model. Differences in the effect of treatment with polidocanol, phenol/phenol benzoate, and polidocanol with doxycycline show that this model displays a dynamic range of tissue responses that allow us to draw comparisons between treatment modalities based on their relative effects. These studies further validate our use of sclerosing agents in biomaterial formulations, and the results allow us to interpret the additive therapeutic effect of encapsulating these agents in electrospun microparticles with bolus and sustained release properties.

### 3.5 Sustained Release Polyester Embolization Particles Enhance Tubal Damage *In Vivo*

**Figure 6:**
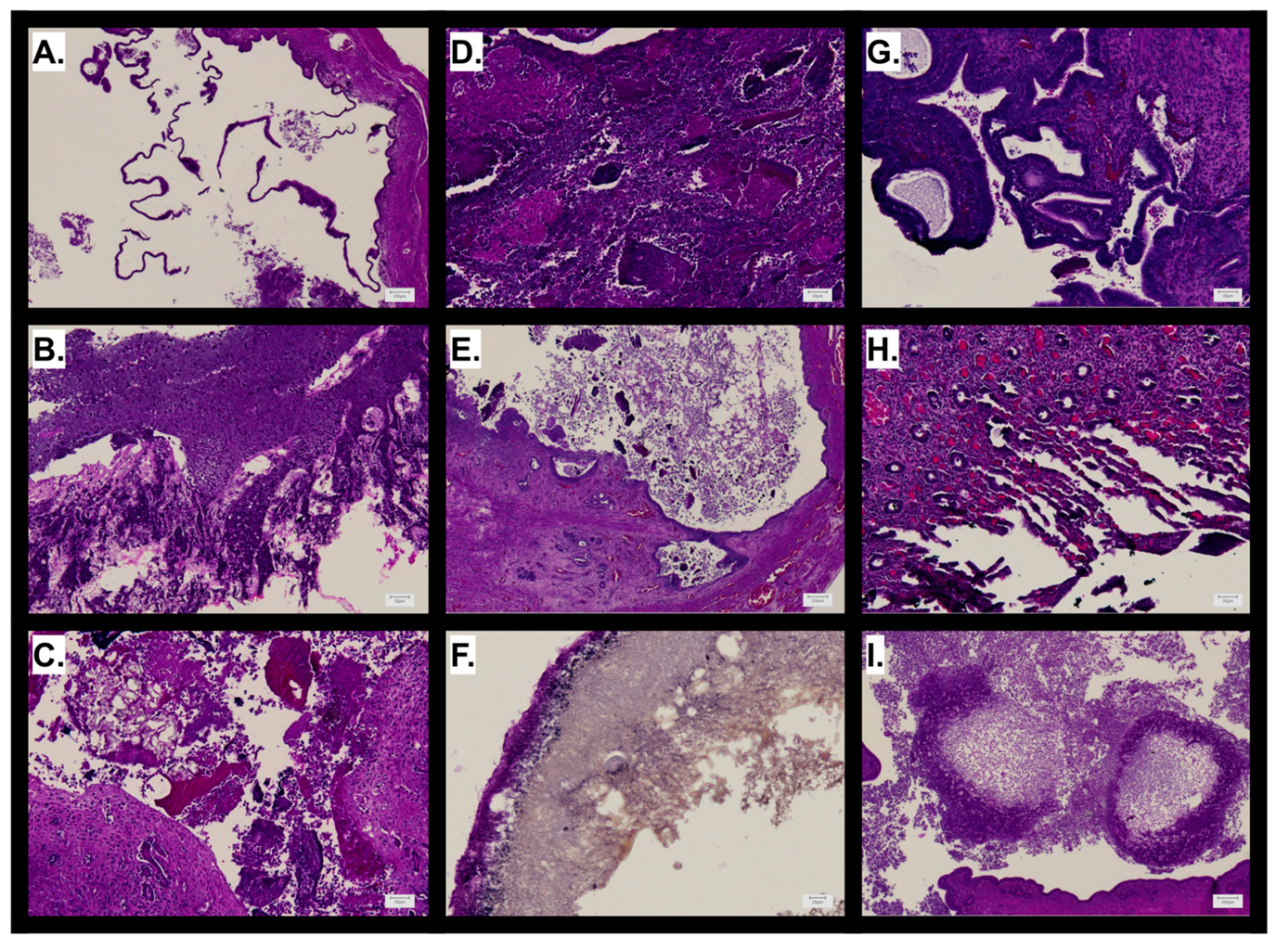
Histological features of guinea pig oviducts and uterine horns after treatment with encapsulated microparticles. Representative images from uterine horns treated with (A, B) 10 mg of drug-free fiber microparticles for 30 days; (C) 20 mg of fiber microparticles loaded with 70 wt % doxycycline instilled with 0.5 mL of 5% PF for 7 days; (D) 20 mg of fiber microparticles loaded with 70 wt % doxycycline instilled with saline for 7 days; (E) 20 mg of fiber microparticles loaded with 70 wt % doxycycline instilled with saline for 30 days; (F, G) 20 mg of fiber microparticles loaded with 30 wt % doxycycline monohydrate instilled with saline for 30 days; (H) 20 mg of fiber microparticles loaded with 80 wt % phenyl benzoate instilled with saline for 7 days; (I) 20 mg of fiber microparticles loaded with 80 wt % phenyl benzoate instilled with saline for 30 days.

We assessed the therapeutic potential of delivering active sclerosing agents encapsulated in polyester microparticles to probe the effects of material potency and acute versus sustained treatment residency. We first investigated the role of polymer materials (vehicle only) in eliciting tissue trauma by instilling drug-free microparticles alone in gelatin capsules. We anticipated that in the low-fluid environment of the uterine horn, degradation of PLGA nanofibers would be slow and acute tissue responses would dampen the sclerosant effect. At 30 days, we observed extensive epithelial detachment and encapsulation of implanted materials (Figure 6A,B). There also appears to be inflammatory cells surrounding the implant, which indicates that polyester fibers alone have active antagonistic properties capable of instigating a cellular response and inflicting long-term damage on the fallopian tubes. Their contribution to acute tissue responses was assessed by testing microparticles containing doxycycline instilled in 5% PF foam and saline. Polyester blend particles loaded with 70 wt % doxycycline were considered bolus release particles as we saw that drug loading above 40 wt % drove rapid drug release from all fiber formulations. Treatment with bolus release doxycycline microparticles and 5% PF resulted in distention of the distal uterine horns accompanied by extensive lumenal residue and a solid yellow discharge containing significant inflammatory cell infiltrate (Figure 6C). Bolus release doxycycline particles inserted with saline, instead of PF, caused slightly less consistent epithelial detachment in all three treated horns but similar inflammatory cell infiltrate and lumenal residue and an overall very similar acute response (Figure 6D). Compared to the treatment group with free doxycycline powder and PF foam control (Figure 5I), the treatment groups with bolus release doxycycline particles (with and without PF) resulted in significantly more inflammation and epithelial damage at 7 days. While the formulated sclerosant is more active than the neat sclerosant, it is unclear whether this is due to release effects of the sclerosant from the polyester fibers or an additive/synergistic effect of the vehicle and sclerosant. Given either mechanism, it is clear that polyester fibers play a necessary role in bolstering the acute tissue response to delivered sclerosing agent.

Finally, we assessed sustained release formulations to examine long-term effects of drug-releasing microparticles. Sustained release microparticles were formulated with 30 wt % doxycycline monohydrate, which is slightly less hydrophilic than the hyclate salt form. While bolus release microparticles showed inflammation but no encapsulation of particle debris at 30 days (Figure 6E), sustained release particles caused fibrous encapsulation of the particle-containing gelatin capsule (Figure 6F) and more extensive inflammation and epithelial distension (Figure 6G). Ultimately, occlusive scarring did not occur in any doxycycline macroparticle treatments groups. However, the emergence of red blood cell infiltrate potentiates a foreign body response (FBR), as contact to blood is the first step of fibrous capsule formation;^42^ therefore, the FBR is a potential downstream response to this treatment group. While sustained release particles elicited more extensive fibrous encapsulation, perhaps due to prolonged residency of polyester fibers, epithelial damage was comparable to that of drug-free particles, suggesting the low dose of doxycycline was insufficient to cause significant epithelial trauma.

The balance between treatment residency and potency was addressed by testing highly loaded phenyl benzoate microparticles. Microparticles containing 80 wt % phenyl benzoate showed first-order sustained release for approximately 25 days *in vitro*, likely due to the high hydrophobicity and crystalline drug form. Treatment with this formulation elicited acute hemorrhage of surrounding tissue at 7 days (Figure 6H) followed by long-term inflammation and fibrous encapsulation of remaining lumenal content, ultimately causing modest occlusive scar tissue formation at 30 days (Figure 6I). These results potentiate polyester phenyl benzoate embolization particles as a viable treatment which may cause tubal blockade at later time points. It is evident that phenyl benzoate is a potent sclerosant; however, it only exerted long-term effects when formulated into electrospun fibers. This is particularly interesting considering the composition of the solid dispersion, which is only 20 wt % polyester blend and 80 wt % drug crystal. One might expect only a modest contribution of the polymer matrix based on this ratio. The mechanism by which fiber encapsulation causes sustained release and durable action of phenyl benzoate remains to be elucidated and may relate to the crystalline state of the drug as it is influenced by electrospinning processing parameters. It is also likely that electrospinning with such a low polymer concentration relative to drug resulted in electrospraying, which would have bound phenyl benzoate crystals into nanocapsules that were compressed together and milled in the micronization process.^44^ In this case, the polymer matrix would contribute to surface area-mediated diffusion of drug into the low-fluid environment. Further investigation into the morphology and dissolution characteristics of supersaturated solid dispersions of hydrophobic drugs is warranted. This sustained release behavior was used to our advantage in this study, wherein phenyl benzoate loaded microparticles were the most promising experimental group.

This is the first study to use guinea pigs as a model for permanent contraception. In contrast to prior studies using polidocanol foam and bolus doxycycline in nonhuman primates, our results show a consistently higher threshold of antagonistic action required to elicit chronic inflammation and fibrotic tissue formation in guinea pigs.^40^ Thus, achieving moderate to successful occlusion of a guinea pig uterine horn may imply more robust occlusion in a higher order model. Testing this strategy in the more sensitive macaque fallopian tubes may better represent its potential for permanent contraception in humans compared to these trials. Our findings that polyester material persists in the oviduct for 30 days, and that fibers themselves elicit an inflammatory response and cause fibrous capsule formation, implicates the foreign body response in addition to anticipated wound healing mechanisms.^43^ As such, this treatment can be considered a hybrid method of occlusion that is, in theory, capable of generating collagen deposition and scar tissue caused by chemical trauma and fibrotic encapsulation of implanted polyester microparticles. Moreover, the foreign body response has been shown to depend significantly on the size and shape of macroparticle (>100 μm) implants.^45^ We propose that delivery of micronized fiber particles of heterogeneous size and shape is an opportune method of optimizing a FBR, as it is likely that some population of particles within a delivered dose elicits maximum fibrosis with respect to dimensionality. To confirm this conclusion, further research must be done to characterize the nature of the immune response to polyester material and candidate sclerosing agents in the fallopian tubes. Hernandez et al. recently showed that the uterus is a tolerogenic environment that dampens a foreign body response; however, the existence of such immune privilege in the immediate utero-tubal junction and the further distal fallopian isthmus warrants investigation.^46^ Optimization of drug loading and release characteristics may better take advantage of the postulated hybrid immune response by instigating stronger acute trauma to initiate wound healing and longer chronic inflammation to facilitate fibrous capsule formation.

A limit of this *in vivo* model is the use of gelatin capsules to isolate particles into the target anatomical location, thus eliminating the variable of particle delivery due to species anatomy. Though the use of capsules is appropriate to test efficacy in theory, it is not directly translatable to a procedure that can be performed in women without a practiced physician, as is the ultimate goal. As such, we conclude that this treatment has significant potential as a nonsurgical permanent contraceptive and warrants further investigation to address delivery and proximal fallopian tube targeting via transcervical catheterization. In future studies, the safety and efficacy of this approach must be rigorously investigated in higher order animals and humans. This work potentiates electrospun microparticles as an effective alternative to repeated administration of drug in bolus form.

## 4 Conclusion

In this work, we investigated drug-eluting polyester microparticles as a biomaterial agent for nonsurgical permanent contraception. We examined the potential of two candidate FDA-approved sclerosing agents, doxycycline and phenyl benzoate, to elicit tissue damage and acute inflammation in explant rhesus macaque fallopian epithelial tissue. We saw that both drugs caused extensive cell infiltration and epithelial trauma which warranted their formulation into a biomaterial drug delivery system. Electrospinning proved an effective method of loading both physiochemically diverse agents with high encapsulation efficiency into polyester nanofibers, thus overcoming limitations of low drug loading seen with other particle formulation strategies. The versatility of blending polymers allowed us to design materials with mechanical characteristics amenable to processing into micronizable nanofiber mats, thereby permitting scalable microparticle production via free-surface electrospinning. Further, we evaluated the release profile of candidate sclerosing agents from nanofibers contingent on several tunable factors, including drug loading, encapsulated agent hydrophobicity, polymer hydrophobicity, and crystallinity, ultimately generating bolus and sustained release particles with potential to elicit acute and prolonged tissue responses. Finally, we validated a guinea pig uterine horn model for tubal occlusion and evaluated the potential of this approach, observing significant acute tissue trauma and sustained inflammation in treatment groups containing nanofibers. We ultimately determined that these materials represent an innovative formulation strategy for generating tunable polymer microparticles that hold significant potential to elicit fibrotic tissue formation and tubal occlusion.

## Supporting Information Available

Puncture force of polyester blends; puncture test properties of polyester blends; glass transition temperature of polyesters; tensile adhesion of polyester blends shows mucoadhesive potential (PDF)
