# Supplementary material for: Drug Eluting Embolization Particles for Permanent Contraception
Source: ACS Biomater Sci Eng. 2022 Jun 24;8(7):2995–3009. doi: 10.1021/acsbiomaterials.2c00357 (PMC9277594; doi:10.1021/acsbiomaterials.2c00357)
Supplement: Supplementary file 1 — ab2c00357_si_001.pdf [file ab2c00357_si_001.pdf]

SUPPORTING INFORMATION

Drug Eluting Embolization Particles for Permanent Contraception

Hannah VanBenschoten, Shan Yao, Jeffrey T. Jensen, Kim A. Woodrow

Supplementary Material – 3 Pages

Supplementary Figure 1

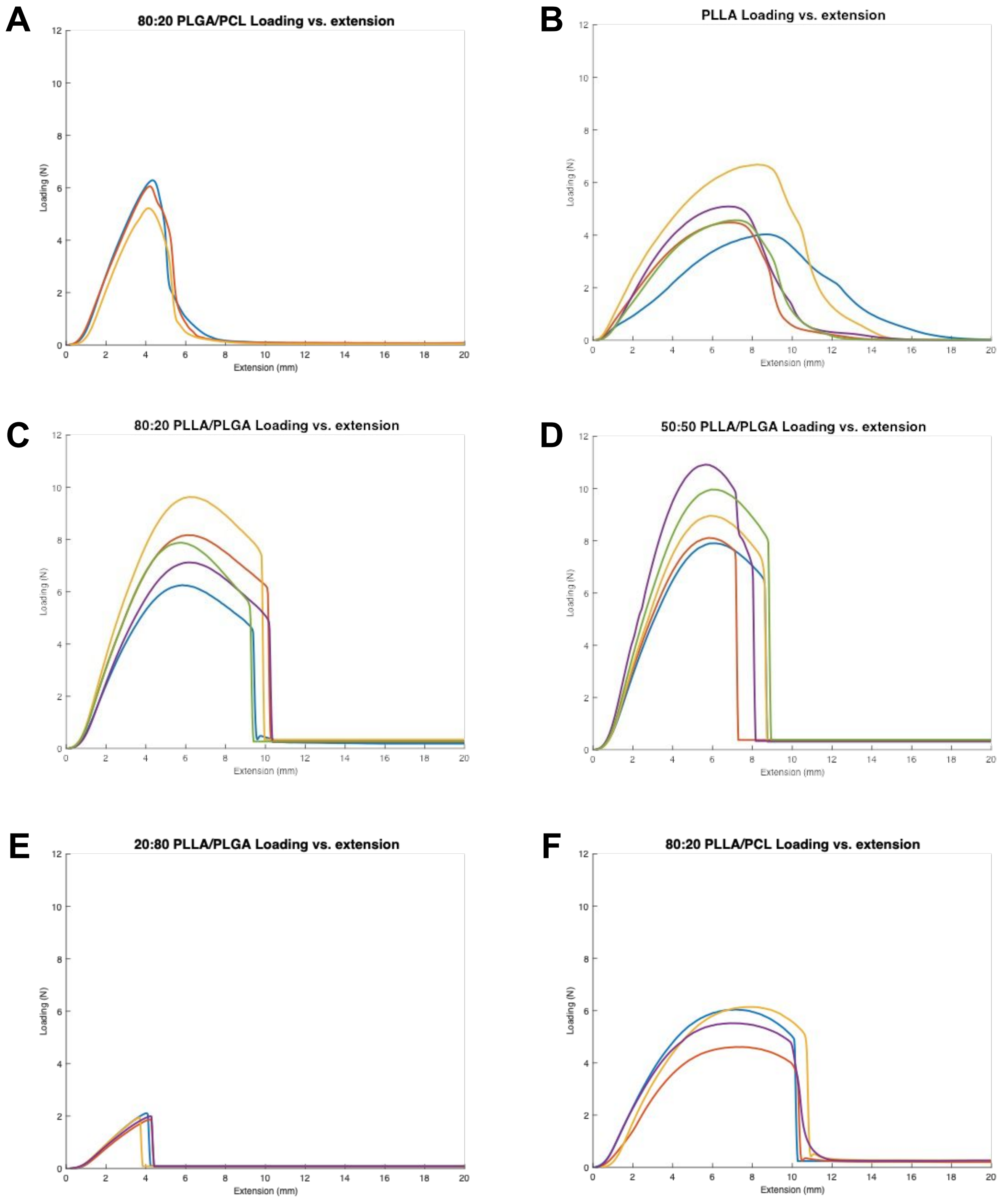

Supplementary Figure 1: Puncture force of electrospun polyester blends. Probe extension (mm) versus load force to puncture (N) of (A) n=3 replicates of 80:20 PLGA/PCL fibers, (B) n=5 replicates of PLLA fibers, (C) n=5 replicates of 80:20 PLLA/PLGA fibers, (D) n=5 replicates of PLLA/PLGA fibers, (E) n=4 replicates of 20:80 PLLA/PLGA fibers, and (F) n=4 replicates of 80:20 PLLA/PCL fibers.

Supplementary Table 1

Puncture Testing of Polyester Blends

Fiber Blend	Burst Load (N)	Puncture Strength (N/mm^2)	Elongation Fraction	Relative puncture strength (N/mm^2)
**80:20 PLGA/PCL**	5.856 (0.562)	0.5871 (0.056)	.2032 (1.313)	2.886 (0.119)
**PLLA**	4.969 (0.103)	0.6228 (0.129)	.5595 (10.73)	1.138 (0.254)
**80:20 PLLA/PLGA**	7.810 (1.258)	0.9788 (0.158)	.3785 (2.725)	2.587 (0.373)
**50:50 PLLA/PLGA**	9.172 (1.270)	1.149 (0.159)	.3699 (1.703)	3.122 (0.535)
**20:80 PLLA/PLGA**	0.7938 (0.097)	0.1990 (0.025)	.2126 (4.020)	0.962 (0.195)
**80:20 PLLA/PCL**	5.577 (0.701)	0.6989 (0.088)	.5255 (3.208)	1.335 (0.195)
**Note: Data represents the mean of n=5 samples (± standard deviation)**				

Supplementary Table 2

Glass and Melting Transition Temperature of Polyesters

Polyester	T_g (°C)	T_m (°C)	References
**PLGA**	40	260	[47]
**PLLA**	59	190	[48, 49]
**PCL**	– 60	60	[31, 40]

Supplementary Figure 2

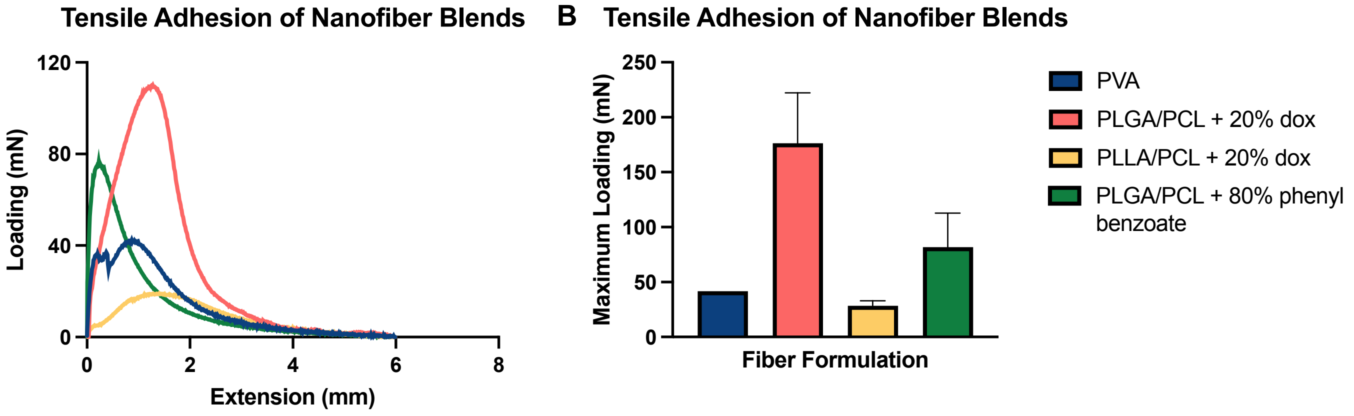

Supplementary Figure 2. Tensile adhesion of polyester blends shows mucoadhesive potential. (A) Loading force (mN) plotted against extension and (B) maximum adhesive force of drug-loaded polyester fiber blends to rehydrated porcine mucin. Results presented as a the mean ± standard deviation of at least n=3 individual fiber samples per condition. One-way ANOVA performed for maximum loading of nanofibers determined that no formulation was significantly less adhesive than PVA (significance defined as $p < 0.05$).
